# *Schistosoma mansoni* granulomas in the skeletal striated muscles in the murine model of neuroschistosomiasis: histological findings

**DOI:** 10.1590/0074-02760190383

**Published:** 2020-05-11

**Authors:** Thiago Andre Alves Fidelis, Geraldo Brasileiro-Filho, Patricia M Parreiras, Paulo Marcos Z Coelho, Neusa Araujo, Marco Vinicius Chaud, Denicezar Angelo Baldo, Nelson Brancaccio dos Santos, José Roberto Lambertucci

**Affiliations:** 1 Universidade Federal de Minas Gerais, Faculdade de Medicina, Departamento de Doenças Infectoparasitárias/Departamento de Anatomia Patológica e Medicina Legal, Belo Horizonte, MG, Brasil; 2 Fundação Oswaldo Cruz-Fiocruz, Instituto René Rachou, Laboratório de Esquistossomose, Belo Horizonte, MG, Brasil; 3 Universidade de Sorocaba, Laboratório de Biomateriais e Nanotecnologia, Sorocaba, SP, Brasil; 4 Pontifícia Universidade Católica de São Paulo, Laboratório de Morfologia e Patologia, Sorocaba, SP, Brasil

**Keywords:** neuroschistosomiasis, muscular lesion, schistosomiasis granuloma

## Abstract

Schistosomiasis mansoni presents many clinical manifestations during migration of schistosomes in their hosts, including diarrhea, hepatomegaly, splenomegaly, liver abscesses, skinlesions, brain tumors and myeloradiculopathy. No lesions have been reported in skeletal striated muscles due to schistosomiasis mansoni in the literature. This short communication reports the histopathological findings on skeletal musculature in a murine model of neuroeschistosomiasis mansoni. Lesions were found in the tongue, masseter muscle, buccinator muscle, digastric muscle and temporalis muscle. Worm recovery was carried out to confirm the infection. We describe here, for the first time in the literature, injuries in the skeletal musculature due to *Schistosoma mansoni* nfection.

Schistosomiasis caused by *Schistosoma mansoni* was first described in Brazil by Pirajá da Silva in 1908. The disease presents atypical complications beyond the gastrointestinal tract, such as upper digestive bleeding, neuroschistosomiasis, glomerulonephritis, and pulmonary hypertension, providing the basis for experimental research.^1^ Muscular lesions associated with schistosomiasis mansoni have never been discussed in the literature. This short communication concomitantly provid the first histopathological documentation of such lesions, reporting granulomas in the striated skeletal musculature containing *S. mansoni* eggs. Animal models may be instrumental to better understand the complex pathogenesis of this unknown disease. To the best of our knowledge, this is the first characterisation of unequivocal skeletal muscles involvement in experimental murine schistosomiasis mansoni.

We infected 25 male mice (*Mus musculus* - *Swiss webster*, weighing between 18 and 20 grams) with 50 LE strain *cercariae* subcutaneously and 25 animals were maintained as controls (uninfected). All animals were followed for 160 days post-infection. At 97 (animal #1), 109 (animal #2) and 146 (animal #3) days post-infection, euthanasia procedures were performed (n = 2/group; infected and uninfected mice, respectively) by CO2 gas chamber, according to guidelines and principles of the Brazilian Council on Animal Care. The protocol was approved by the local Institutional Animal Care Committees at the Federal University of Minas Gerais and at the René Rachou Research Institute [Oswaldo Cruz Foundation (Fiocruz), State of Minas Gerais, Brazil]. The *ex vivo* samples had a catheter placed into the right heart and perfused by a fixative solution of 10% paraformaldehyde (PFA). Worm recovery was carried out as per the technique prescribed by Pellegrino and Siqueira.^2^ Experiments were performed on a 7T magnetic resonance scanner (MRI System 7T/210 ASR Horizontal Bore Magnet, Agilent Technologies, Palo Alto, CA, USA). *Ex vivo* brain images were obtained using 3D T1 Gre (TR/TE: 370 ms/5 ms, Matrix: 128 x 96 x 96, FA: 35º, Nex: 13, Fov:20 × 15 × 15 mm, acquisition time: 12h 18min), coronal Multi Echo (TE/TR: 3,000/9 ms, 3 Echos, Nex:30, Matrix: 128 × 128, Fov:15 × 15 mm, Slices: 30, Slice Thickness:0.5 mm, no Gap, acquisition time:3h 12 min). After imaging, brain and skull were immersed in 7% nitric acid for decalcification. After one day (24 h), the whole skull was sectioned in 3 mm thick (frontal slices) and placed in 7% nitric acid for 24 h for complete decalcification. After that, the fragments were sectioned in 1.1 mm thick slices, each one placed in a paraffin block (10-11 blocks for each animal). Serial 4 µm sections (obtained from 50 µm intervals between each) from all paraffin blocks were stained with haematoxylin and eosin (H&E). Light microscope was used to search for any morphological lesion, especially *Schistosoma* eggs and/or granulomas. The right hemisphere of each animal’s skull was stained with Nankin® ink for identification (Figure).

**Figure:**
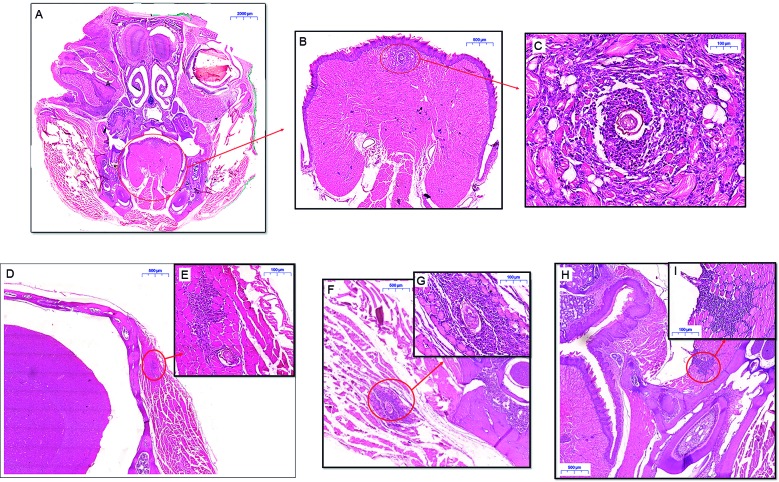
Histological images. (A) Frontal cut of the skull haematoxylin and eosin (H&E); (B) Detail of the fusiform lesion in the tongue (H&E); (C) Granuloma formation is noted with *Schistosoma mansoni* egg in the centre (H&E); (D) A lesion of the temporal muscle (H&E); (E) Granuloma and *S. mansoni* egg in the temporal muscle (H&E); (F) Injury of the masseter muscle (H&E); (G) Detail of the lesion with granuloma around *S. mansoni* egg (H&E); (H) Granuloma in the buccinator muscle (H&E); (I) Granuloma formation without *S. mansoni* egg (H&E).

In 25 *Swiss webster* mice subcutaneously infected with 50 *cercariae* of the *S. mansoni* (LE strain), three mice (animals #1, #2 and #3) presented neurological manifestations such as spinning, hemiparesis and ataxia. Recovery of worms in animals #1, #2, #3 confirmed infection. Histology confirmed lesions in the brain associated with *S. mansoni* eggs in all three infected mice. During histopathological study of the brain, we incidentally found periovular granuloma formation with infiltration of macrophages, neutrophils, eosinophils, lymphocytes and occasional giant cells in the tongue, masseter muscle, buccinator muscle, digastric muscle and temporalis muscle (Figure), undoubtedly reproducing muscular infection by schistosomiasis mansoni in three of the 25 infected mice (12%). Animals from the control group presented no changes. Magnetic resonance imaging (MRI) revealed abnormalities in the brain of the all three studied infected mice. Prior MRI analysis had not identified muscular lesions.

The model adopted in this study demonstrated granulomas in the encephalon and skeletal muscles of infected mice, being the first characterisation of unequivocal skeletal striated muscles involvement in a murine model of neuroschistosomiasis mansoni. The experimental model is widely used for the reproduction of the severe presentation of schistosomiasis mansoni, including hepatic fibrosis, nephropathy, pulmonary hypertension and neuroschistosomiasis. *Swiss webster* mice infected with *S. mansoni* present periovular granulomas distributed in the hepatic parenchyma with portovenous lesions similar to Symmers fibrosis in humans.^3^ Pneumopathies in *S. mansoni* infected mice due to arteritis caused by eggs and granulomas produces pulmonary hypertension as observed in human disease. Sections of the lungs revealed many granulomata and central necrosis.^4^ Fugiwara et al.^5^ reported severe glomerulonephritis observed in four out of 10 infected *BXSB* female mice, exposed to 10 *S. mansoni cercariae*, examined histologically. Fidelis et al.^6^ ^,^ ^7^ reproduced experimental brain schistosomiasis using *Swiss webster* mice infected subcutaneously. Eggs and granulomas were found dispersed in the brain, brainstem and cerebellum. The model of neuroschistosomiasis mansoni also identified eggs and granulomas in the bulbar conjunctiva, lacrimal gland, choroid and corneoscleral limbus in some infected mice.

Lambertucci et al.^8^ have previously reported a case of a 28-year-old man who has been living in an endemic area for schistosomiasis (Ribeirão das Neves, Minas Gerais, Brazil). The patient and his wife decided that he would undergo a vasectomy for contraception. Twenty days following vasectomy, a semen analysis was performed in a Newbauer chamber and a few mature *S. mansoni* eggs were observed. Lopes et al.^9^ identified *S. mansoni* eggs in the seminal vesicle in a 62-year-old male with suspected adenocarcinoma prostate diagnosis. Radical prostatectomy was performed and histopathological examination confirmed the diagnosis of adenocarcinoma and *S. mansoni* eggs in the seminal vesicle. Andrade-Filho et al.^10^ related a case of a 14-year-old boy with popular cutaneous lesions in periumbilical region bilaterally. These lesions were presumed to represent cercarial dermatitis. The other case, a 32-year-old man, with presumed diagnosis of micronodular sarcoidosis, lichen planus or cutaneous schistosomiasis. Histopathological examination of the skin lesions, in both cases, revealed granulomas with *S. mansoni* eggs.^10^ This brief communication describes and reports, for the first time in the literature, injuries in the skeletal musculature of the definitive host, in this case *Swiss webster* mice, due to *S. mansoni*. Interestingly, these lesions could not be demonstrated on prior MRI scans, likely due to their small size. Saeed et al.^11^ reported a case of a 52-year-old patient presented with asymptomatic disseminated myocysticercosis in the right femural quadríceps, chest and pelvis skeletal muscles. Lesions were confirmed by cytology and histopathology. Pallav et al.^12^ reported an unusual case of cysticercosis in the right soleus muscle that presented as acute calf pain and difficulty in walking. Enzyme-linked immunosorbent assay (ELISA) for IgG antibodies against *Taenia solium* was positive, which confirmed the diagnosis. Chaurasia et al.^13^ related a case of 22-year-old woman with recurrent swelling and pain on her right cheek for three years. The histology confirmed myocysticercosis in the right masseter muscle. Reddi et al.^14^ and Bhat et al.^15^ reported similar cases of myocysticercosis lesions presenting with pain and limitation of mouth opening in masseter muscle diagnosed by imaging exams.

Schistosomiasis of the skeletal muscles is an extremely unsuspected disease that must be related to human patients from endemic areas who develop symptoms of disseminated muscle pain. Further studies with this experimental model may help shed light to the pathophysiology of skeletal muscles changes associated with this fascinating disease. The authors hope that this brief communication will publicise this new ectopic form of schistosomiasis.
